# Transcriptional Analysis-Based Alterations Affecting Neuritogenesis of the Peripheral Nervous System in Psoriasis

**DOI:** 10.3390/life12010111

**Published:** 2022-01-13

**Authors:** Dóra Romhányi, Kornélia Szabó, Lajos Kemény, Endre Sebestyén, Gergely Groma

**Affiliations:** 1 Department of Dermatology and Allergology, University of Szeged, H-6720 Szeged, Hungary; romhanyidora9411@gmail.com (D.R.); szabo.kornelia@med.u-szeged.hu (K.S.); kemeny.lajos@med.u-szeged.hu (L.K.); 2 Hungarian Centre of Excellence for Molecular Medicine-University of Szeged Skin Research Group (HCEMM-USZ Skin Research Group), University of Szeged, H-6720 Szeged, Hungary; 3 Eötvös Loránd Research Network, MTA-SZTE Dermatological Research Group, H-6720 Szeged, Hungary; 4 1st Department of Pathology and Experimental Cancer Research, Semmelweis University, H-1085 Budapest, Hungary; endre.sebestyen@gmail.com

**Keywords:** psoriasis, cutaneous nervous system, axon development, myelination

## Abstract

An increasing amount of evidence indicates the critical role of the cutaneous nervous system in the initiation and maintenance of psoriatic skin lesions by neurogenic inflammation. However, molecular mechanisms affecting cutaneous neurons are largely uncharacterized. Therefore, we reanalyzed a psoriatic RNA sequencing dataset from published transcriptome experiments of nearly 300 individuals. Using the Ingenuity Pathway Analysis software, we associated several hundreds of differentially expressed transcripts (DETs) to nervous system development and functions. Since neuronal projections were previously reported to be affected in psoriasis, we performed an in-depth analysis of neurite formation-related process. Our *in silico* analysis suggests that SEMA-PLXN and ROBO-DCC-UNC5 regulating axonal growth and repulsion are differentially affected in non-lesional and lesional skin samples. We identified opposing expressional alterations in secreted ligands for axonal guidance signaling (RTN4/NOGOA, NTNs, SEMAs, SLITs) and non-conventional axon guidance regulating ligands, including WNT5A and their receptors, modulating axon formation. These differences in neuritogenesis may explain the abnormal cutaneous nerve filament formation described in psoriatic skin. The processes also influence T-cell activation and infiltration, thus highlighting an additional angle of the crosstalk between the cutaneous nervous system and the immune responses in psoriasis pathogenesis, in addition to the known neurogenic pro-inflammatory mediators.

## 1. Introduction

Psoriasis is a chronic inflammatory skin disease affecting approximately 1–3% of the human population worldwide. It is characterized mainly as an abnormal skin reaction to various internal and external stimuli, leading to keratinocyte hyperproliferation and chronic immunological responses [1]. Despite the large amount of work, the exact pathomechanism of psoriasis remains unclear. Therefore, a deeper understanding of the disease-causing alterations is important to develop new treatment options that not only treat the existing symptoms but also interfere with their development.

In psoriasis, the macroscopically healthy-looking non-lesional (NL) skin already carries alterations that in combination with various abiotic and biotic stimuli lead to the appearance of symptoms [2,3]. One of the widely known characteristics of the NL skin is the Köbner phenomenon, the development of lesions in response to mechanical provocations or stress [4] due to elevated immune response and increased keratinocyte proliferation [5,6]. External, potentially dangerous stimuli are not only sensed by keratinocytes but also by cutaneous neurons, among other cells. Skin cells become activated by these insults, produce pro-inflammatory cytokines [7], and may also activate and modulate the neuronal functions of nociceptors [8]. An example of this is an altered thermosensation in psoriatic tissues [9]. 

Peripheral nervous system (PNS) abnormalities resulting in the loss of sensory abilities could lead to the remission of psoriatic lesions, including, I. superficial cutaneous nerve injury [10], II. poliomyelitis-associated flaccid paralysis [11], III. loss of intercostobrachial nerve function [12], IV. permanent severance of the left lateral cutaneous nerve [13], V. traumatic unilateral brachial plexus palsy [14], VI. loss of finger sensation due to peripheral denervation [15] and VII. partial axonal and demyelinating neuropathy [16]. Damages affecting the central nervous system (and thereby also the PNS) e.g., hemiparesis [17] and hemiplegia [18] or stroke [19] were reported to cause the clearance of psoriatic plaques at the neuronal dysfunction-affected peripheral areas. Moreover, in cases when the nerve injury-associated anesthesia was only temporary, psoriatic symptoms reappeared following nerve function recovery [14]. These case reports were recently summarized in depth by Bi Qin and colleagues [20] and by Tian Hao Zhu and coworkers [21].

Apart from the nervous system-related injuries, several case series showed near-complete remission of psoriatic lesions following botulinum toxin treatment [22,23] that further supports the role of the nervous system both in the formation, as well as in the maintenance of psoriatic plaques. In a psoriasiform animal model, botulinum toxin treatment was suggested to exert its effect through the inhibition of neuropeptides [24]. In 1986, researchers suggested the influence of cutaneous neurons and neuro-immune factors in the pathogenesis of psoriasis [25]. Since then, numerous studies indicated the role of neuropeptides both in the inflammatory and the proliferative processes in psoriasis pathogenesis. As a result, we may consider psoriasis, at least in part, as a neurogenic inflammatory disease [26]. Studies reported increased expression of several neuropeptides in the lesional (L) skin, including CGRP (calcitonin gene-related protein) [27,28], NGF (nerve growth factor) [29], SP (substance P) [30,31], VIP (vasoactive intestinal peptide) [32]. Apart from their neural functions, these molecules also display pro-inflammatory activities and thereby may contribute to inflammation [33], highlighting an important role of the nervous system in psoriasis pathomechanism.

The majority of psoriatic patients are troubled by itch at their L skin [34,35]. In these areas, neurogenic pro-inflammatory mediators, e.g., CGRP, NGF, and SP can contribute to itching (pruritus) development [36,37,38]. Thus, patients may also suffer from aching, burning, cramping, stinging, tenderness, and tingling at the L areas [39], suggesting that cutaneous neuronal sensation mechanisms are affected at multiple levels.

While our knowledge of how neurons affect the immune system is continuously increasing, little is known about how the cutaneous nervous system itself is affected in psoriasis. Several large-scale studies, including proteomics [40,41], RNA microarray [42], and high throughput sequencing (HTS) [43,44], GWAS [45], and DNA methylation profiling [46] analyses have been performed to gain a deeper insight into the pathomechanism of the disease [47]. However, our knowledge of the involvement of the peripheral and the cutaneous nervous system in psoriasis remains limited. 

Therefore we took advantage of the transcriptome sequencing results of three major published psoriatic datasets and reanalyzed the combined data by using the Ingenuity Pathway Analysis software for downstream enrichment analysis [43,44,48]. Using an unbiased annotation, we found 347 and 885 differentially expressed transcripts (DETs) in relation to the nervous system in NL and L skin samples, respectively. These DETs were associated with nervous system development and functions, in particular, with neuritogenesis-regulating mechanisms. This may seem peculiar, knowing that the cell body of neurons are not located in the skin. However, earlier studies indicate that massive RNA transport and translation take place in the axons that are located at cutaneous tissues [49,50]. Therefore, we decided to focus on these mechanisms by analyzing neuritogenesis-related alterations in depth.

## 2. Materials and Methods

### 2.1. Criteria for Combining the Transcriptome Sequencing Data of from Three Published Psoriatic Datasets

To identify general alterations in psoriasis and to avoid any potentially non-disease related associations and differences, randomly engaged individuals of chronic plaque psoriatic patient and healthy donors were involved in the three studies [43,44,48] of which the combined database was generated from. For inclusion criteria no preference of gender, age (apart from >18), or Psoriasis Area Severity Index scores (min. 1% of total body surface area) was put forward in any of the three studies, of which the RNA sequencing data were collected from. Similarly, skin punch biopsies (6 mm) were collected from various regions of the body (hip, buttlock, thigh, back, arm, flank, abdomen, elbow). A wash out period of 1 week for patients on topical anti-psoriatic treatments and 2 weeks for those on any systemic anti-psoriatic treatments was set as general criteria prior to biopsy collection.

### 2.2. RNA Sequencing Data Processing

The RNA sequencing datasets from three papers were uniformly reprocessed [43,44,48]. We downloaded the data from SRA (Sequence Read Archive, https://www.ncbi.nlm.nih.gov/sra, accessed on 15 November 2021) with study ID accession numbers SRP035988, SRP050971, and SRP055813 using SRA-tools (version 2.9.2, https://github.com/ncbi/sra-tools, accessed on 15 November 2021) and reprocessed all available samples. We quantified transcript level expression using Kallisto [51] (version 0.43.0) and the full GENCODE [52] v27 transcriptome annotation, available at https://www.gencodegenes.org (accessed on 15 November 2021). Kallisto was run with the following options: --bias --single -l 120 -s 20 -b 100.

### 2.3. Differential Expression Analysis

Transcript-level length-scaled TPM (Transcripts Per Million) expression estimates from Kallisto were imported into the R statistical environment (version 3.4.3, https://www.r-project.org; accessed on 15 November 2021), using the tximport [53] package (version 1.6.0). The data were TMM (trimmed mean of M-values) normalized and voom transformed. We used edgeR [54] (version 3.20.9) for the TMM normalization and the voomWithQualityWeights() function from limma [55,56] (version 3.34.9) for the voom transformation. We decided to use voomWithQualityWeights() to combine transcript observation-level weights with sample-specific weight, as we did not want to discard samples with lower quality, but preferred to downweigh them in the analysis. Limma was also used to test for differential expression between lesional and non-lesional, lesional and healthy, or non-lesional and healthy sample groups. A linear model was fitted with the limma lmFit function, and the moderated t-statistics was calculated with the eBayes function. Transcripts were defined as differentially expressed if they had an FDR [56,57] (false discovery rate) corrected *p*-value < 0.05 and an absolute log2 fold-change larger than 1.

### 2.4. Functional Annotation, Enrichment Analysis, and Statistics

Differentially expressed transcripts (DETs) from NL vs. H and L vs. H comparison were analyzed using Ingenuity Pathway Analysis (IPA) software (IngenuityH Systems, www.ingenuity.com; accessed on 15 November 2021) to identify pathways that are enriched. DET sets were mapped to the HUGO gene symbols within IPA software and those that did not map to any HUGO gene were discarded. For “Diseases and Biological functions” annotation, the *p*-value was calculated using Fisher’s exact test [58] to measure the significance of DET enrichment of a given pathway. For the Gene Ontology enrichment analysis and visualization (Gorilla) tool, the enrichment analysis *p*-value was calculated according to the mHG or HG model [59]; *p*-value correction for multiple testing was done according to the Benjamini and Hochberg method [60] (FDR correction). Enrichment was defined as: (b/n)/(B/N), where N: total number of genes, B: total number of genes associated with a given specific GO term, n: number of genes in the top of the user’s input list or in the target set when appropriate, b: number of genes in the intersection.

## 3. Results

### 3.1. Peripheral Nervous System-Associated Transcript Expression Alterations in Psoriasis

Based on our transcriptome analysis, 2681 transcripts showed altered expression level in the NL and healthy (H) skin comparison (Supplementary Table S1A, whereas the number of transcripts with altered expression in L vs. H skin was 12314 (Supplementary Table S1B).

Ingenuity Pathway Analysis (IPA) software identified DETs coded by 347 and 885 genes in association with nervous system development and function in NL and L skin, respectively (Supplementary Table S1C,D). These DETs are predicted to affect neuronal morphogenesis, including neuritogenesis, which represented the most specific group in the analysis (Table 1 and Supplementary Table S1E,F).

### 3.2. Differentially Expressed Transcripts Affecting Axon-Related Alterations in Non-Lesional and Lesional Psoriatic Skin

Since only neurites penetrate the skin, we wanted to gain further insight into how neuron projections are likely to be affected in the skin. For this, we performed gene ontology (GO) functional enrichment analysis using neuron projection GO:0043005 as a background in Gorilla (Gene Ontology enrichment analysis and visualization tool; accessed on 15 November 2021.) on the neuritogenesis-associated DETs from the original IPA analysis. This analysis revealed biological processes linked to the regulation of neuron projection development and the semaphorin-plexin signaling pathway. According to our results, these pathways are likely to be affected already in the NL skin, and to a greater extent in L samples, as suggested by a higher number of DETs in the latter group (Table 2 and Supplementary Table S1G,H).

In addition, neuron projection morphogenesis, development, and guidance (Table 3 and Supplementary Table S1I,J) were predicted to be affected only in psoriatic lesions (Table 4 and Supplementary Table S1K,L). Among axon formation-associated regulatory processes, negative regulation of axonogenesis and axon guidance are predicted to be affected in psoriatic lesions (Table 4 and Supplementary Table S1K,L).

Axon formation is strongly associated with Schwann cell myelination in the peripheral nervous system. Despite that functional enrichment analysis did not reveal any associated processes, skin tissue expression analysis (tissues.jensenlab.org; accessed on 15 November 2021) integrated into the STRING database (version:11.5; accessed on 15 November 2021) revealing certain associations. Four molecules (MBP, MPZ, PMP22, and EGR2) out of the DETs coded by 347 genes in NL were assigned to Schwann cells (BTO:0001220, 4 of 6 molecules), and another four (MBP, MPZ, PMP22, and RTN4) to myelin (BTO:0000894, 4 of 6 molecules). A similar analysis also pointed out four (MBP, MPZ, EGR2, and PRX) Schwann cell-associated molecules in L skin samples (out of the DETs coded by 885 genes), while myelin-related molecules were MBP, MPZ, PLP1, and RTN4 (Supplementary Table S1M). Our analysis suggests that a common molecule that emerges is RTN4 (also known as Nogo), thus myelin-associated inhibitory regulation of axon formation via RTN4 appears to be a common mechanism both in NL and L skin samples.

### 3.3. Semaphorin-Plexin Signaling, An Important Regulator of Axon Formation, Is Differentially Affected in Non-Lesional and Lesional Psoriatic Skin

**Figure 1:**
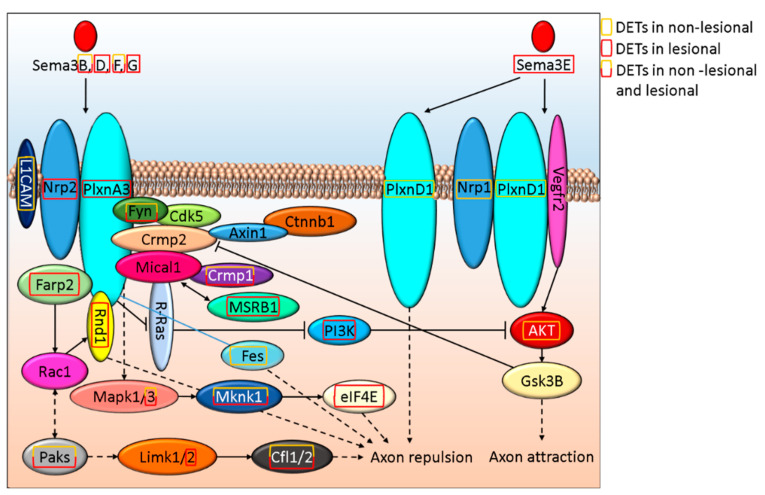
*In silico* model of how Sema3 signaling alterations regulate axon morphogenesis in NL and L psoriatic skin.

Since both IPA and GOrilla enrichment analysis suggested that Semaphorin-Plexin signaling is affected (Semaphorin Neuronal Repulsive Signaling Pathway: *p*-value_NL vs. H_ = 1.52E-03 and *p*-value_L vs. H_ = 1.45E-02 and Table 2., respectively) in psoriasis pathogenesis, we analyzed these pathways in depth. Type 3 semaphorins (Sema3) play a role in neurite formation by regulating axon attraction and repulsion. Among the Sema3 family members that inhibit axon extension, we found DETs coded by Sema3B and Sema3F genes both in NL and L skin, while in L skin, we also detected Sema3D, Sema3E, and Sema3G expression (Figure 1 and Supplementary Table S1N). Sema3A is not affected by DETs in NL or L skin. Among semaphorin3 receptors and coreceptors, L1CAM, Nrp1and PlxnD1 are only affected by DETs in NL skin, while in L samples gene expressional differences are associated with Nrp2 and PlxnA3 (Figure 1.). Transcripts of downstream signaling molecules Fyn, Crpm1, Mapk3, Mknk1, and Paks are differentially expressed both in NL and L skin. Fes and AKT expression are altered only in NL, while DETs of eIF4E, Farp2, Limk2, MsrB1, PI3K, and Rnd1 are present in lesions (Figure 1). These abnormalities may suggest that axon repulsion and the negative regulation of axon attraction is likely to be highly affected in L in contrast to NL skin, where PI3K-mediated negative regulation of axon attraction does not seem to play a role when compared to H skin samples.

**Figure 2:**
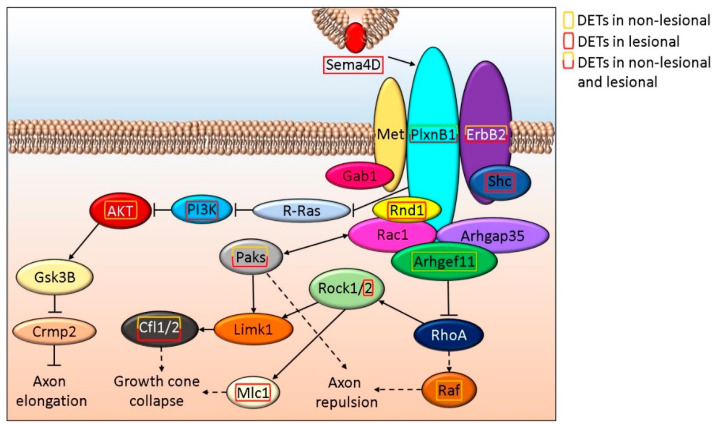
Schematic *in silico* model of the role Sema4D signaling play in axon elongation/repulsion in NL and L skin.

Sema4D is important in axon regeneration not only by modulating axon elongation but also by inhibiting neuron myelination [61]. SEMA4D encoding DETs are present in L but not in NL skin. Sema4D cell surface receptors (PlxnD1 and ErbB2), as well as downstream signaling proteins (Paks, Cfl1, and Cfl29) expression are altered in NL and L skin. Whereas in NL skin, AKT, Arhgef11, and RAF, while in L samples Mlc1, PI3K, Rnd1, Rock2, and Shc are affected by DETs (Figure 2 and Supplementary Table S1O).

Sema6A and 6D gene expression is affected in lesions that share receptors of Sema3A, as well as CSPG, the receptor of Sema5A. These alterations may also affect axon repulsion. The schematic *in silico* model of the potential crosstalk between Sema3-Sema4-Sema5-Sema6 signaling is shown in Supplementary Figure S1 and Table S1P, while Supplementary Figure S2 and Table S1Q shows the interaction of Sema4 and Sema7A signaling, which only affected in L samples.

### 3.4. ROBO-DCC-UNC5 Signaling Regulates Axon Formation and Differentially Affected in Non-Lesional and Lesional Psoriatic Skin

**Figure 3:**
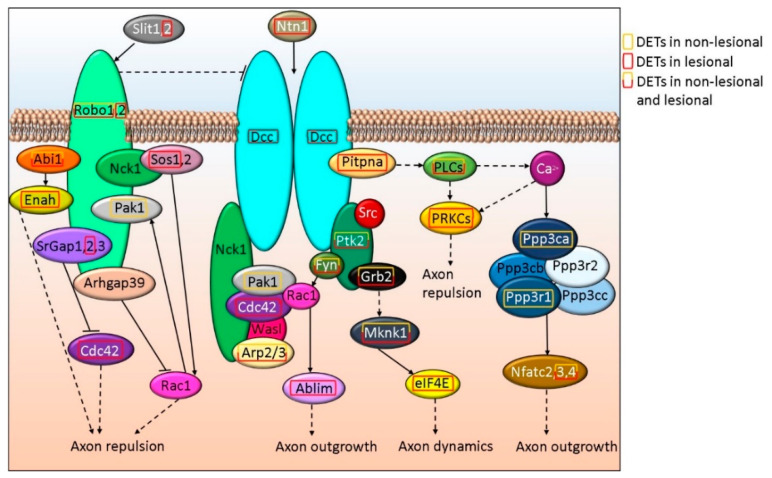
Schematic *in silico* model of axon outgrowth/repulsion regulation via Robo-DCC signaling-related alterations in NL and L skin.

**Figure 4:**
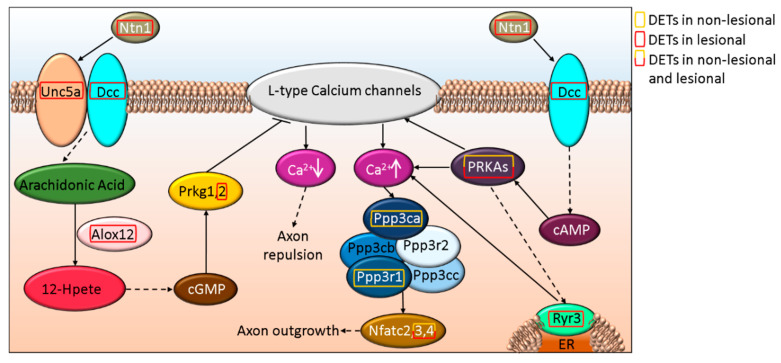
Schematic *in silico* model of axon outgrowth/repulsion regulation by UNC5A-DCC signaling-related alterations in NL and L skin.

Axon dynamics is also regulated through Slit and Ntn signaling via Robo and Dcc, respectively. Slit and Ntn signaling via Robo and Dcc were found as part of the general canonical signaling pathway term Axonal Guidance Signaling that also included Wnt5a and semaphorins and were suggested to be affected both in NL and Lesional skin (*p*-value_NL vs. H_ = 3.21E-5 and *p*-value_L vs. H_ = 5.03E-06, respectively). SLIT2 and its receptor ROBO2 are affected only in L skin, while ROBO1 expression is altered in NL and L samples (Figure 3 and Supplementary Table S1R). The expression of NTN1, as well as its receptors DCC (Figure 3) and UNC5A (Figure 4 and Supplementary Table S1S) are affected in L but not in NL skin, where only some of the downstream proteins may be differentially expressed.

### 3.5. Disturbed WNT5A Signaling Potentially Affect Cutaneous Axon Growth in Psoriasis

**Figure 5:**
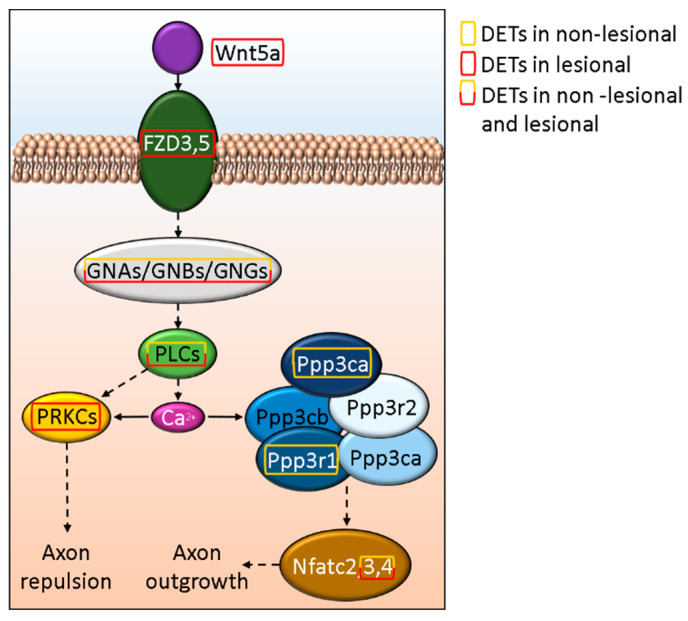
*In silico* model of the effect of Wnt5a signaling on axon growth and retention in psoriasis.

We found that WNT5A is affected in psoriatic lesions, and the FZD3 and FZD5 receptor-mediated (also affected in L skin) signaling pathway may play a role in axon growth/repulsion (Figure 5 and Supplementary Table S1T). In contrast, we only found DETs of downstream molecules in the NL skin, and these were mostly affecting axon outgrowth (Figure 5.).

## 4. Discussion

Psoriasis is a chronic inflammatory skin disease where interleukin (IL)-17 is the major driver of inflammatory responses [62]. Studies, including the imiquimod-induced psoriasis-like skin inflammation models in mice [63], suggest that the peripheral nervous system may have a role in the initiation and maintenance of the inflammatory and hyperproliferative responses through the release of neuropeptides [26,30]. Cutaneous nerves can activate dermal dendritic cells’ IL-23 production, and IL-23 triggers IL-17 expression and release by T cells [63]. Drugs targeting IL-17 and IL-23 are, as a matter of fact, are some of the most effective drugs available to treat psoriasis [64,65]. Therefore, peripheral nervous system-related abnormalities could be important to understand the pathomechanism of psoriasis.

During the reanalysis of public RNA-sequencing data, we found that neuritogenesis might be disturbed in psoriatic patients. For example Semaphorin-Plexin signaling cascades regulate various features of neuronal projection formation-related processes [66]. Semaphorins were originally identified as neuronal and axon growth guidance molecules. Today it is clear that the superfamily of semaphorins counting over 20 members of soluble extracellular and cell surface transmembrane signaling proteins, can modulate the development and function of several organs, including the cardiovascular [67], immune [68,69], and the nervous system [70], among others [71,72]. Despite their massive role in innate immune responses and inflammation [73], we have limited data about the semaphorins’ involvement in psoriasis pathogenesis [74,75,76], but no information on axon formation-related processes in the context of this disease. Since neuritogenesis might be affected [77] in psoriasis, it is not surprising that DETs of semaphorins (Semas) were identified in our study, given their clear role in axon guidance. Most of the molecules, like SemaB and SemaF, were implicated in both axon attraction or repulsion [78]. These antagonistic functions may be due to the differences in their local concentrations, and/or the receptor repertoire on the interacting cells. For example, Sema3E stimulates axon growth of PLXND1 and NRP1 expressing neurons, but when PlexinD1 is expressed without NRP1, Sema3E has an opposite effect [79]. In addition, Sema3E interaction with its co-receptor VEGFR2 may also stimulate axon extension [80]. Despite that VEGFR2 was not affected in our analyzed dataset, the expression of other Sema receptors, including NRP1, NRP2, PLXNA3, PLXNB1, PLXNB3, PLXND1, as well as L1CAM and ERBB2 were differentially expressed in psoriatic samples. The decrease in Sema3D could negatively influence both the numbers and the branching of peripheral axons [81]. Interestingly, the expression of this molecule was affected in lesions, which may be a reason why the number of neurites and axonal branching is reduced in psoriatic patients [77]. NRP1was suggested to play in the pathomechanism of psoriasis by several studies in context with keratinocyte proliferation and differentiation, angiogenesis and lymphangiogenesis among others (reviewed by Sunhyo Ryu and colleagues [76]). 

Class IV semaphorins are thought to be transmembrane proteins [73]. Sema4D presented at the surface of the cells is known to influence axon regeneration, and its overexpression can inhibit neuron myelination [61]. Sema4D is also expressed by various immune cells, including T cells, and could modulate dendritic cell functions [82]. In psoriasis, T cells infiltrate not only to the dermis, where they may interact with dermal dendritic cells, but also the epidermis to contact with Langerhans cells. Sema4D was also suggested to induce keratinocyte mediated inflammatory responses in psoriasis [75]. Moreover, myelination of neurites is the least pronounced in the epidermis, where Sema4D(+) T cells may interrupt the myelination processes, and thereby inhibit axon regeneration. In line with this concept, we found several major myelin-associated proteins [83], including MBP, MPZ, PMP22, and RTN4 with altered expression in psoriatic lesions. RTN4 (also known as NOGOA) is a myelin-associated inhibitor of axon growth and regeneration following nerve injury [84] and may contribute to the reduction of neurites [77] in lesions.

We also found SLIT2 and its receptor ROBO1, previously shown to be expressed by both axons and Schwann cells, and ROBO2 that is mainly expressed by axons in mice [85] in the lesional samples of our dataset. Schwann cell-expressed NTN1, which participates in axon regeneration following nerve injury [86], is also affected in psoriatic lesions. NTN1 can also influence neutrophil, macrophage, and T-cell infiltration [87]. In addition, dendritic cell-originated Sema4A might play a role in the activation of both Th1 and Th17 cells in the neuroinflammatory demyelinating autoimmune disease, multiple sclerosis [88,89]. This molecule is also affected by DETs in psoriatic patients.

In the nervous system, Sema4B plays a role in synapse formation and maintenance and might influence post-synaptic density [90]. We found altered expression of this molecule only in NL skin. In addition, Sema4B could inhibit basophil-mediated Th2 skewing [91] and contributes to the developing Th1/Th2 imbalance in psoriasis [92]. Apart from this, circular SEMA4B RNA may decrease the effect of IL-1β through Wnt signaling [93]. This pathway may also influence axon growth/repulsion via WNT5A (and its receptors FZD3 and FZD5) that we found to be affected in psoriatic lesions which is in line with previous observation [94]. It may also act as a suppressor of axonal regeneration [95], and at the same time, facilitate CXCL12-CXCR4-mediated T-cell infiltration [96], with the latter being known to be important in chronic inflammatory skin diseases [97].

Therefore, we suggest that the dysregulation in 12 different semaphorins and some of their main receptors and co-receptors could contribute to the abnormal neuron projection formation described earlier in psoriasis [77]. Semaphorin signaling could also greatly influence other major hallmarks of psoriasis, for example the innate immune and inflammatory processes [73]. Therefore, our study can highlight an additional angle of the crosstalk between the neuro-immune system, which might be another important factor in the psoriasis pathomechanism, in addition to the neurogenic pro-inflammatory mediators. Our study provides a strong base and novel directions in psoriatic research since due to the discrepancy that differentially expressed mRNAs do not necessarily correlate with protein expression level, therefore future studies are required to analyze these correlations and to validate the cellular association of DETs.

It is important to note that the vast majority of semaphorin signaling cascades, as well as SLIT-ROBO and NTN-DCC signaling, exert their effect through the small GTPase RAC1 [84]. This molecule not only connects the cutaneous nervous system and the immune cells but also keratinocytes, where it can influence proliferation, differentiation, and innate immune processes [98]. Based on these features, RAC1 is likely to be an important molecule in psoriasis.

RAC1 is also known as a Ras-related C3 botulinum toxin substrate 1, as it is the primary target of botulinum toxin.

Our results together with previous observations provide an explanation why botulinum toxin treatment of patients could be so effective and argues for its more extensive clinical application in psoriasis therapy.

## Figures and Tables

**Table 1:** Functional annotation of nervous system related DETs in non-lesional and lesional psoriatic skin. (H: healthy, L: lesional, NL: non-lesional skin).

Categories	Functions	Comparison	*p*-Value	Number of Molecules
Nervous System Development and Function	Morphology of nervous system	NL vs. H	4.11E-17	236
		L vs. H	5.28E-32	637
Nervous System Development and Function, Neurological Disease	Abnormal morphology of nervous system	NL vs. H	4.95E-13	188
		L vs. H	2.80E-20	495
Nervous System Development and Function, Tissue Morphology	Morphology of nervous tissue	NL vs. H	1.11E-12	165
		L vs. H	5.25E-22	439
Nervous System Development and Function, Organismal Development, Tissue Development	Morphogenesis of nervous tissue	NL vs. H	4.70E-10	144
		L vs. H	4.46E-22	405
Cell Morphology, Cellular Assembly and Organization, Cellular Development, Cellular Function and Maintenance, Cellular Growth and Proliferation, Nervous System Development and Function, Organismal Development, Tissue Development	Neuritogenesis	NL vs. H	5.26E-10	142
		L vs. H	6.62E-22	399
Cell Morphology, Cellular Development, Cellular Growth and Proliferation, Nervous System Development and Function, Organismal Development, Tissue Development	Morphogenesis of neurons	NL vs. H	6.60E-10	143
		L vs. H	6.85E-22	403
Cellular Development, Cellular Growth and Proliferation, Nervous System Development and Function, Tissue Development	Development of neurons	NL vs. H	1.12E-09	177
		L vs. H	2.14E-24	517

**Table 2:** Gene ontology (GO) functional enrichment analysis of DETs associated with neuritogenesis in non-lesional and lesional skin. (H: healthy, L: lesional, NL: non-lesional skin).

GO Term	Description	Comparison	*p*-Value	FDR q-Value	Enrichment (N, B, n, b)
GO:0010975	Regulation of neuron projection development	NL vs. H	1.98E-4	2.74E-2	1.72 (1442, 229, 139, 38)
		L vs. H	6.66E-10	2.79E-7	1.64 (1594, 260, 389, 104)
GO:0045664	Regulation of neuron differentiation	NL vs. H	2.68E-4	3.35E-2	1.67 (1442, 249, 139, 40)
		L vs. H	6.63E-11	4.07E-8	1.64 (1594, 285, 389, 114)
GO:0071526	Semaphorin-plexin signaling pathway	NL vs. H	4.09E-4	4.07E-2	5.19 (1442, 12, 139, 6)
		L vs. H	2.3E-5	1.37E-3	2.96 (1594, 18, 389, 13)

**Table 3:** Gene ontology (GO) functional enrichment analysis of DETs associated with neuritogenesis reveals neuron projection-related biological processes in lesional but not in non-lesional skin. (H: healthy, L: lesional).

GO Term	Description	Comparison	*p-*Value	FDR q-Value	Enrichment (N, B, n, b)
GO:0048812	Neuron projection morphogenesis	L vs. H	2.94E-10	1.43E-7	1.96 (1594, 138, 389, 66)
GO:0097485	Neuron projection guidance		8.37E-7	9.17E-5	1.78 (1594, 124, 389, 54)
GO:0031175	Neuron projection development		9.31E-7	9.74E-5	1.68 (1594, 159, 389, 65)
GO:0010976	Positive regulation of neuron projection development		3.15E-6	2.54E-4	1.69 (1594, 141, 389, 58)
GO:0010977	Negative regulation of neuron projection development		9.99E-5	4.76E-3	1.74 (1594, 87, 389, 37)

**Table 4:** Gene ontology (GO) functional enrichment analysis of DETs associated with neuritogenesis reveals axon formation-related biological processes only in lesional psoriatic skin. (H: healthy, L: lesional).

GO Term	Description	Comparison	*p*-Value	FDR q-Value	Enrichment (N, B, n, b)
GO:0050770	Regulation of axonogenesis	L vs. H	6.36E-7	7.32E-5	1.89 (1594, 102, 389, 47)
GO:0007411	Axon guidance		8.37E-7	9.06E-5	1.78 (1594, 124, 389, 54)
GO:1902668	Negative regulation of axon guidance		8.36E-5	4.2E-3	3.00 (1594, 15, 389, 11)
GO:0048843	Negative regulation of axon extension involved in axon guidance		9.41E-5	4.56E-3	3.15 (1594, 13, 389, 10)
GO:0050771	Negative regulation of axonogenesis		2.61E-4	1.07E-2	2.00 (1594, 45, 389, 22)
GO:0008045	Motor neuron axon guidance		6.07E-4	2.21E-2	2.73 (1594, 15, 389, 10)
GO:0048841	Regulation of axon extension involved in axon guidance		6.07E-4	2.2E-2	2.73 (1594, 15, 389, 10)
GO:1902667	Regulation of axon guidance		9.35E-4	3.19E-2	2.50 (1594, 18, 389, 11)

## Data Availability

Only publicly available data was used in the study (Sequence Read Archive, https://www.ncbi.nlm.nih.gov/sra; study ID: SRP035988, SRP050971, and SRP055813). Processed data (differential expression analysis) is provided as supplementary material (Table S1).

## Supplementary Materials

The following supporting information can be downloaded at: https://www.mdpi.com/article/10.3390/life12010111/s1, Figure S1: *In silico* model of the potential crosstalk between Sema3-Sema4-Sema5-Sema6 signaling in NL and L psoriatic skin; Figure S2: *In silico* model of the interaction between Sema4 and Sema7A signaling in NL and L psoriatic skin; Table S1: Peripheral nervous system-associated transcript expression alterations in psoriasis.
