# Supplementary material for: Transcriptional Analysis-Based Alterations Affecting Neuritogenesis of the Peripheral Nervous System in Psoriasis
Source: Life (Basel). 2022 Jan 13;12(1):111. doi: 10.3390/life12010111 (PMC8778302; doi:10.3390/life12010111)
Supplement: Supplementary file 1 [file life-12-00111-s001.zip › life-1489764- supplementary-for XML.pdf]

life

Supplementary material of Transcriptional Analysis-Based Alterations Affecting Neuritogenesis of the Peripheral Nervous System in Psoriasis

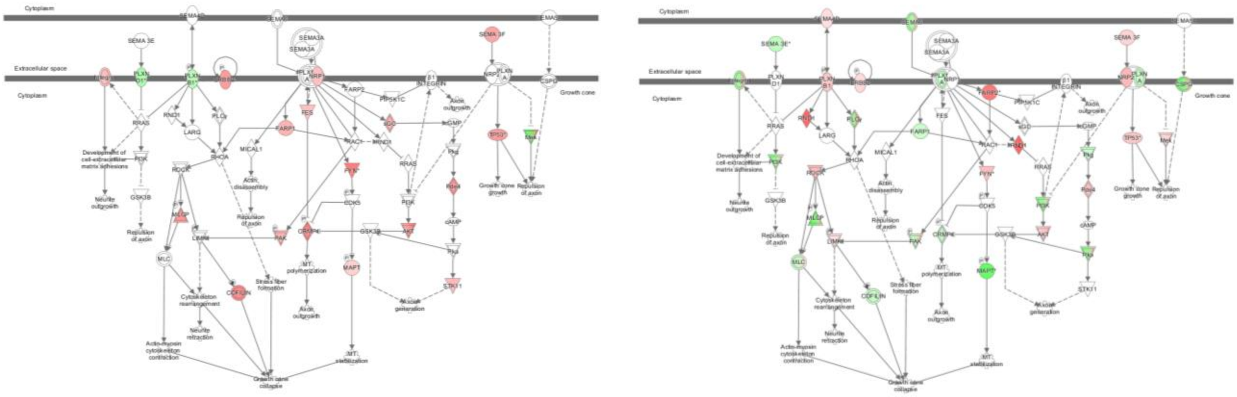

Figure S1. *In silico* model of the potential crosstalk between Sema3-Sema4-Sema5-Sema6 signaling in NL (left panel) and L (right panel) psoriatic skin. DETs are colored according to the extent of the difference compared to healthy samples. Green color depicts decreased and red increased expression levels. (QIAGENs Ingenuity Pathway Analysis software was used to generate the image.)

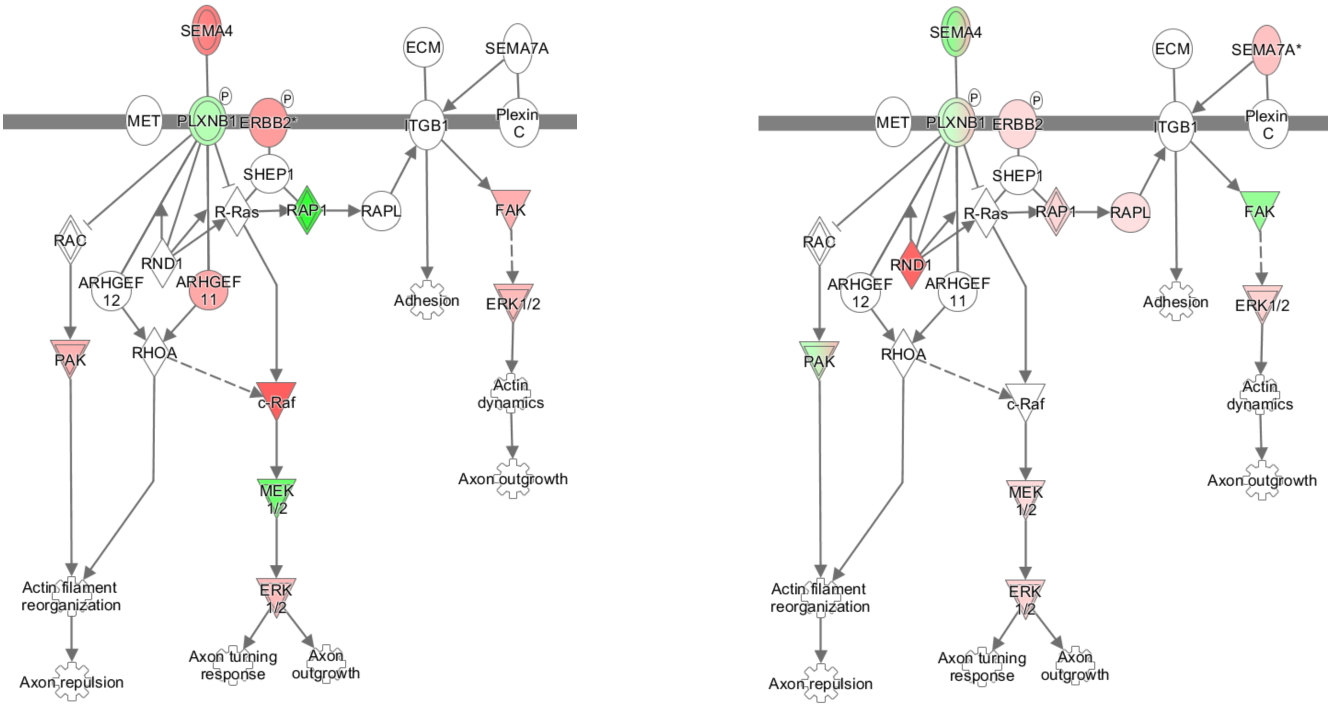

Figure S2. *In silico* model of the interaction between Sema4 and Sema7A signaling in NL (left panel) and L (right panel) psoriatic skin. DETs are colored according to the extent of the difference compared to healthy samples. Green color depicts decreased and red increased expression levels. (QIAGENs Ingenuity Pathway Analysis software was used to generate the image.)
